# Efficient Catalytic Degradation of Methyl Orange by Various ZnO-Doped Lignin-Based Carbons

**DOI:** 10.3390/molecules29081817

**Published:** 2024-04-17

**Authors:** Zhihao Tang, Yonggang Yang, Weiqi Wei

**Affiliations:** 1 Jiangsu Co-Innovation Center of Efficient Processing and Utilization of Forest Resources, Nanjing Forestry University, Nanjing 210037, China; tangzhihao@njfu.edu.cn; 2 School of Environmental & Resource Science, Shanxi University, Taiyuan 030006, China; m18352353192@163.com

**Keywords:** LC/ZnO composites, methyl orange, photocatalytic degradation

## Abstract

Herein, a series of ZnO-doped lignin-based carbons (LC/ZnO) were successfully prepared from different types of lignin and used for methyl orange (MO) photocatalytic degradation. The apparent morphology, internal structure, and photoelectric properties of prepared LC/ZnO composites and their effects on subsequent MO photocatalytic degradation were investigated by various characterization techniques. The results showed that the LC/ZnO composites that were prepared in this work mainly consisted of highly dispersed ZnO nanoparticles and lignin-based carbon nano-sheets, which were beneficial for subsequent photogenerated electrons and holes formation, dispersion, and migration. The MO could be significantly degraded with various ZnO-doped lignin-based carbons, especially over the LC_SL_/ZnO, and the maximum degradation rate was 96.9% within 30 min under the simulated 300w sunlight exposure. The experiments of free radical elimination showed that the photocatalytic degradation of MO over LC/ZnO were a result of the co-action of multiple free radicals, and h^+^ might play the predominant roles in MO degradation. In addition, the pH of the solution had little effect on MO degradation, and the MO could be effectively degraded even in an alkaline solution of pH = 12.0. The cycling experiments showed that the prepared LC/ZnO had a good stability for MO photodegradation, especially for LC_SL_/ZnO, even after 5 times recycling, and the degradation rate of MO only dropped from 97.0% to 93.0%. The research not only provided a fundamental theory for the efficient photocatalytic degradation of MO by LC/ZnO composites, but also offered a new insight into lignin valorization.

## 1. Introduction

With the rapid evolution of industry, ambient damage and excessive waste of resources were gradual impediments to adherence to a sustainable development path. The random discharge of industrial waste had caused a significant blow to the ecological environment. And industrial wastewater was a major source of environmental pollution, especially for low concentration industrial wastewater, because it was difficult to treat it by traditional treatment methods [1,2,3]. As a result, photocatalysis technology was born as a valid way to solve the existent contamination and energy crunch, which could completely transform industrial dyeing wastewater into non-toxic and harmless substances without leaving any secondary pollution. In addition, the used catalyst could be recycled, which reduced the waste of resources and improved reusability of resources [4,5]. Industrial wastewater was common in life, such as the paper black liquor produced in the pulp papermaking process and various dyes made in the printing and dyeing process. Therefore, photocatalytic technology was used as a sharp weapon to solve the problem of industrial wastewater pollution and wastewater recycling in order to maximize the protection of the ecological environment. At present, many types of photocatalysts (e.g., ZnO, TiO_2_, SnO_2_, etc.) [6], have been developed. Among them, ZnO had a relatively weak response to visible light, and its photogenerated electrons and holes were easy to recombination, which was also easily available and non-toxic. However, ZnO could only employ ultraviolet light, which was only part of the sunbeam, and the photoelectric ionophore generated was also easy to polymerize again, which led to low photocatalytic efficiency. In addition, ZnO was prone to photocatalytic corrosion and polymerization during the photocatalytic procedure, which brought about poor recyclability. In order to solve this problem, carbon materials were usually used to solve the separation and migration between photogenerated electrons and holes [7,8,9]. In industrial production, it was necessary to deliberate multiple considerations (such as economic factors, environmental factor, etc.). More importantly, there was an increasing focus on taking full advantage of biomass resources. Lignin is a kind of aromatic polymer with three-dimensional network crosslinking, which is abundant and renewable. It contains numerous benzene ring structures with a carbon content of up to 60%, so it is a perfect carbon precursor [10]. There was an increasing employment of lignin derivatives (e.g., guaiacol extracted from industrial lignin was translated into phenol by hydrodeoxygenation on a carbon-supported MoO_2_ catalyst) [7]. Regularly shaped hollow microspheres were prepared by a self-assembly method using sodium lignosulfonate (SL) as a carbon source for adsorption and degradation experiments, the excellent role of lignin carbon as a carrier in photocatalysis was also introduced [11]. According to previous studies, natural ilmenite sand was used as a source of iron and titanium, and sucrose was used as a source of carbon to successfully prepare TiO_2_-Fe_3_C-Fe-Fe_3_O_4_ composites dispersed on graphite carbon framework. The fabricated composites effectively adsorb methylene blue via π–π interactions. They can effectively photodegrade methylene blue under sunlight [12,13]. Furthermore, the prospect of lignin-based catalysts for photocatalytic degradation of industrial dyes and their catalytic mechanism were also expounded in previous studies [7,14].

In industrial production, it was common goals to bring due economic returns and create an environment-friendly and resource-saving society [15,16], and high-value utilization of lignin helped create this condition [17,18]. Therefore, the high-value utilization of lignin had become the focus of attention [19]. The rise in lignin-based materials made industrial and academic circles see its development momentum. For example, its adsorbent field, photocatalytic degradation field, supercapacitor electrode material field, and lithium-ion battery field also showed its excellent performance [20,21,22]. Therefore, many researchers have further validated the application of lignin-based carbon materials in catalysis by compounding a variety of lignin-based carbon materials with the aforementioned ZnO and comparing their photocatalytic activities [23,24,25].

This work aimed to investigate the efficiency of photocatalytic degradation of MO by using different ZnO-doped lignin-based carbons. In the formation process of LC/ZnO composite, Simultaneous lignin carbonization during the decomposition of the precursor ZnC_2_O_4_ into ZnO, and the ZnO nanoparticles were uniformly dispersed in the three-dimensional network structure of lignin-based carbon nano-sheets. The apparent morphology, internal design, and photoelectric properties of prepared LC/ZnO composites and their effects on subsequent MO degradation were investigated. In addition, the effects of various degradation conditions (such as solution pH, catalyst dosage, etc.) on MO degradation and the potential mechanism for MO photocatalytic degradation over LC/ZnO as well as the stability of prepared LC/ZnO composite for MO photocatalytic degradation were also deeply investigated and discussed.

## 2. Characterization and Analysis

### 2.1. Micromorphology and Microstructure of Various Prepared Catalysts

**Figure 1:**
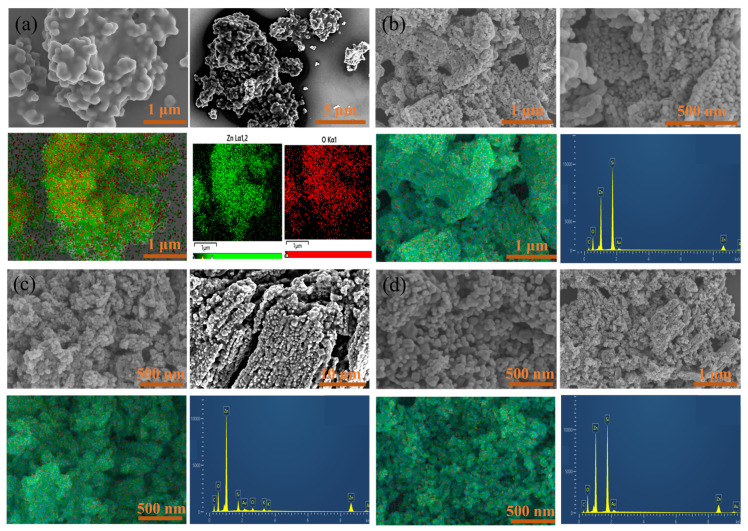
SEM and EDS images of ZnO (**a**), LC_AL_/ZnO (**b**), LC_OL_/ZnO (**c**), and LC_SL_/ZnO (**d**).

Figure 1 displays the SEM and EDS images of ZnO and various ZnO-doped lignin-based carbons. Figure 1a shows that the diameter of ZnO nanoparticles was about 200 nm to 500 nm. However, the accumulation of pure ZnO nanoparticles were severe. Figure 1b shows the accumulation phenomenon of LC_AL_/ZnO nanoparticles was significantly improved, the ZnO was evenly distributed on LC, and its diameter size was reduced to about 100–200 nm. The elemental composition and content of LC_AL_/ZnO had been analyzed by EDS. LC_AL_/ZnO was composed of three main elements: C, Zn, and O, which further showed that the LC_AL_/ZnO composite was successfully prepared, among which the Si element was due to the employment of silicon wafers to stick to the conductive glue during the Cold Field Emission Scanning Electron Microscope experiment. Figure 1c shows that the LC_OL_/ZnO particles were well dispersed and the size between the particles was only about 50-100 nm, which showed good dispersion. Figure 1d shows that LC_SL_/ZnO particles were also well dispersed and the size between the particles was only about 80–150 nm; however, the LC_SL_/ZnO particles were better dispersed and more regularly distributed, which showed the best dispersion.

**Figure 2:**
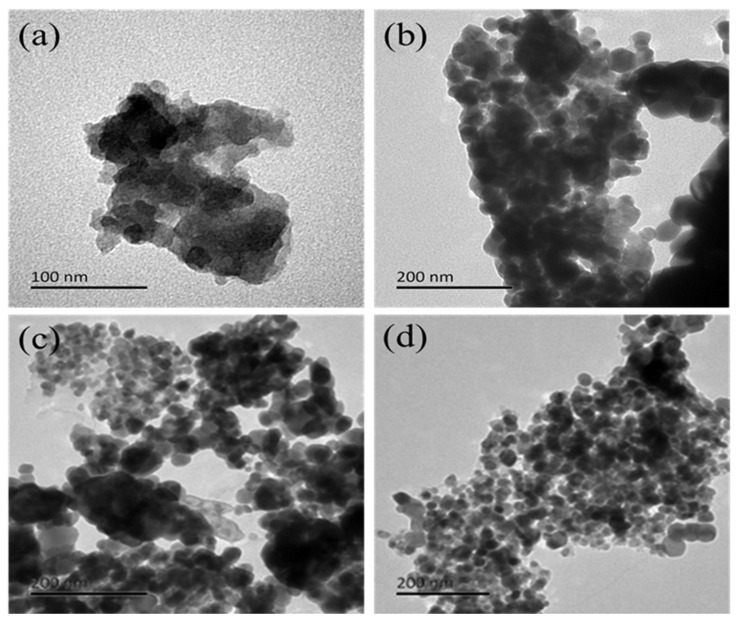
TEM images of ZnO (**a**), LC_AL_/ZnO (**b**), LC_OL_/ZnO (**c**), and LC_SL_/ZnO (**d**).

Figure 2 displayed the TEM images of ZnO and LC_AL_/ZnO, LC_OL_/ZnO, and LC_SL_/ZnO. It was clear that the pure ZnO nanoparticles exhibited a serious accumulation phenomenon (Figure 2a). In comparison with pure ZnO, LC_AL_/ZnO, LC_OL_/ZnO, and LC_SL_/ZnO had better dispersion, which attached well to lignin carbon. The various ZnO-doped lignin-based carbons had smaller particle sizes than ZnO. But beyond that, a good binding of LC_AL_, LC_OL_, and LC_SL_ nano-sheets to ZnO nanoparticles could be seen. In the formation of various ZnO-doped lignin-based carbons, the formation of AL/ZnC_2_O_4_, OL/ZnC_2_O_4_, and SL/ZnC_2_O_4_ precursors was a critical step. The ZnC_2_O_4_ precursor decomposed at high temperature to produce CO_2_ gases; CO_2_ gases as pore-forming agents would make the carbon nano-sheets appear loose and porous, which was conducive to ZnO loading on the lignin carbon nano-sheets. Lignin with a three-dimensional structure as a carbon precursor was conducive to the dispersion of ZnO [26,27,28]. Therefore, the degree of dispersion was the most noticeable in Figure 2d, in which the degree of dispersion of LC_SL_/ZnO was the best, which was further verified for its catalytic effect.

**Figure 3:**
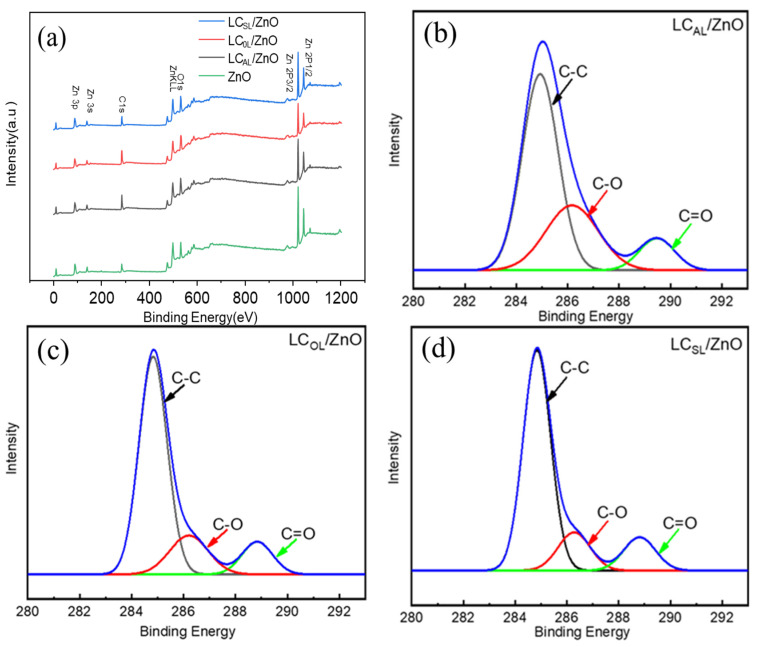
XPS spectra of ZnO and LC_AL_/ZnO, LC_OL_/ZnO, and LC_SL_/ZnO. (**a**) Wide scan; (**b**) C1s spectra of LC_AL_/ZnO; (**c**) C1s spectra of LC_OL_/ZnO; (**d**) C1s spectra of LC_SL_/ZnO.

The elemental composition and bonding modes of ZnO, LC_AL_/ZnO, LC_OL_/ZnO, and LC_SL_/ZnO were characterized by XPS. As shown in Figure 3a, three characteristic peaks of C1s, O1s, and Zn2p with binding energies around 284.6 eV, 536.3 eV, and 1022.4 eV, respectively, which were observed both in full scan of ZnO and various ZnO-doped lignin-based carbons, indicating the ZnO and various ZnO-doped lignin-based carbons prepared in this work contained a large amount of Zn, C, and O elements. However, the C peak in ZnO was mainly due to the carbon in the detection device, and a similar phenomenon was also reported in a previous study [29]. The binding energy at 284.9 eV, 286.1 eV, and 288.8 eV of the C1s spectrum in Figure 3b corresponds to the C-C, C-O, and C=O in LC_AL_/ZnO sample [10]. The C1s spectrum of LC_AL_/ZnO was similar to that of other carbon-based carriers and the position of the peak was not shifted by the use of different lignin; this indicated that the valence state of C would not change during the process of carbonization [7,21].

**Figure 4:**
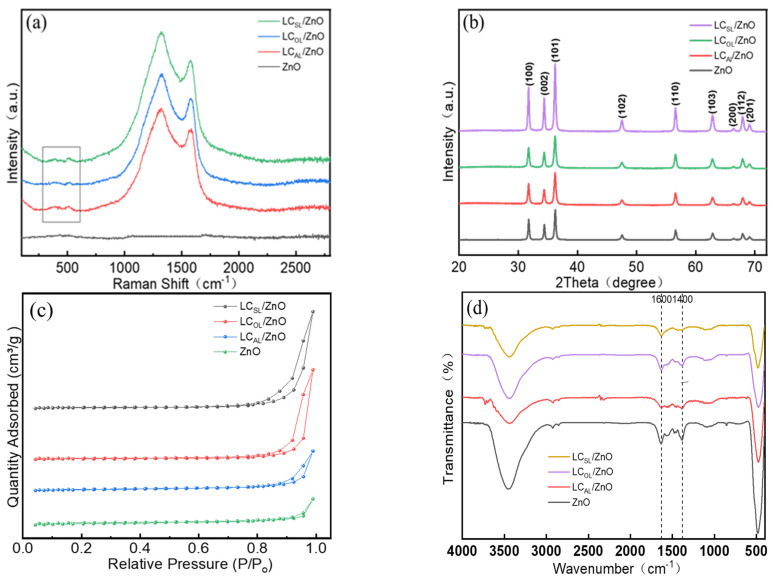
The Raman spectra (**a**); XRD pattern (**b**); N_2_ adsorption−desorption isotherms (**c**); and FT−IR spectra (**d**) of ZnO, LC_AL_/ZnO, LC_OL_/ZnO, and LC_SL_/ZnO.

Raman spectroscopy was chosen to gain more insight into the structure of ZnO, LC_AL_/ZnO, LC_OL_/ZnO, and LC_SL_/ZnO, and the results are shown in Figure 4a. There were two weak bulges in the spectrum of the pure ZnO sample around 400 cm^−1^, and they were generally related to the characteristic peaks of ZnO according to previous reports [30,31]. The degree of disorder of carbon structure and the vibrations of Sp^2^ hybridized carbon atoms led to the formation of D and G bands, respectively. In addition, the I_D_/I_G_ ratio was used to reflect the relative concentration of localized defects in carbon domains. The ratio of I_D_/I_G_ of the prepared various ZnO-doped lignin-based carbons were close to 1.25, similar to previous reports using various carbon and ZnO composites [32,33,34,35,36]. Moreover, the degree of graphitization also could be reflected by the I_D_/I_G_ ratio, different carbon materials, they had the similar values, which indicated that the prepared various ZnO-doped lignin-based carbons had the approximate degree of graphitization. Figure 4b displays the XRD result. As was shown, the XRD pattern of various ZnO-doped lignin-based carbons were almost the same as that of pure ZnO, which indicated that the type of lignin and its co-heating with ZnC_2_O_4_ at high temperatures did not affect the formation of the ZnO crystal structure, and similar phenomena have been reported [1]. 

N_2_ adsorption–desorption was used to examine the specific surface area of ZnO and various ZnO-doped lignin-based carbons LC_AL_/ZnO, LC_OL_/ZnO, and LC_SL_/ZnO, as illustrated in Figure 4c. The N_2_ adsorption–desorption curves of ZnO and LC_AL_/ZnO, LC_OL_/ZnO, and LC_SL_/ZnO exhibited a type-IV isotherm with a H_4_ hysteresis loop at P/P_0_ = 0.8~1.0 according to IUPAC adsorption isotherm classification, indicating the existence of abundant mesopores and macropores in ZnO and LC_AL_/ZnO, LC_OL_/ZnO, and LC_SL_/ZnO, which were in accordance with the average pore diameters shown in Table S1. And the specific surface areas, total pore volume, and average pore diameter of ZnO and LC_AL_/ZnO, LC_OL_/ZnO, and LC_SL_/ZnO are shown in Table S1. The calculated specific surface area of LC_AL_/ZnO, LC_OL_/ZnO, and LC_SL_/ZnO were significantly greater than that of ZnO, presumably contributing to improved photocatalytic performance. The photocatalytic effect of various ZnO-doped lignin-based carbons were significantly stronger than ZnO, which may be due to their large specific surface area and the ability to transport charge carriers [37,38]. Figure 4d displays the changes in the functional groups of lignin before and after the high temperature carbonization of ZnO and various ZnO-doped lignin-based carbons. The broad absorption band of 400–600 cm^−1^ in the ZnO and various ZnO-doped lignin-based carbon samples were mainly caused by the telescopic vibration of Zn-O-Zn and O-Zn-O bonds. The absorption peaks at 1600 cm^−1^ and 1400 cm^−1^ of various ZnO-doped lignin-based carbon samples were primarily attributed to the telescopic vibration of C=C double bonds in the benzene ring skeleton.; as a result, the photocatalytic activity was also greatly enhanced [39,40]. Moreover, the characteristic peaks of oxygen-containing functional groups of the various ZnO-doped lignin-based carbons were significantly reduced, indicating that the carbonization was successfully finished. 

### 2.2. Optical Properties of Various Prepared Catalysts

**Figure 5:**
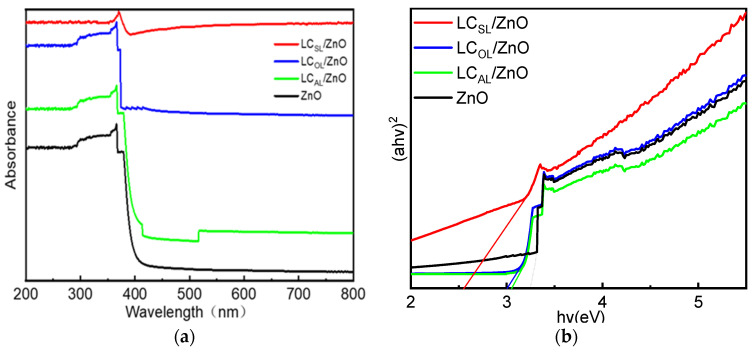
UV-vis absorption spectra of ZnO and LC_AL_/ZnO, LC_OL_/ZnO, and LC_SL_/ZnO.

As shown in Figure 5a, UV spectroscopy was employed to measure the optical properties of ZnO, LC_AL_/ZnO, LC_OL_/ZnO, and LC_SL_/ZnO. The absorption strengths of all catalysts were very high in UV regions within wavelengths less than 400 nm, which indicated that the photocatalysts synthesized in this work have good UV light responsiveness. The various ZnO-doped lignin-based carbons exhibited better UV absorption than the ZnO, mainly due to the C=C and C=O double bonds in the benzene ring structure [41]. In addition, the various ZnO-doped lignin-based carbons had relatively high absorption intensity in visible light caused by the background absorption of lignin carbon, which echoed the black background of the ZnO composites of different lignin carriers [42]. Moreover, the bandgap energy (Eg) could be calculated from (αhv)^2^ versus the photon energy (hv) as the following formula:

a = B_d_(hv – Eg)^1/2^/hv (1)

As shown in Figure 5b, the bandgap energies were calculated from tangent to intercept to be approximately 2.65 eV for ZnO, 3.0 eV for LC_AL_/ZnO, 3.16 eV for LC_OL_/ZnO, and 3.52 eV for LC_SL_/ZnO. Figure 5b shows that the absorption edge of the various ZnO-doped lignin-based carbons were red-shifted, and this phenomenon was conducive to improving the photocatalytic degradation efficiency. The bandgap energy reflected the ease of electron jump from the valence band to the conduction band in semiconductor materials. The increase in bandgap energy indicated that the photogenerated electrons were transferred to the lignin-based carbon, which significantly improved the photocatalytic activity.

**Figure 6:**
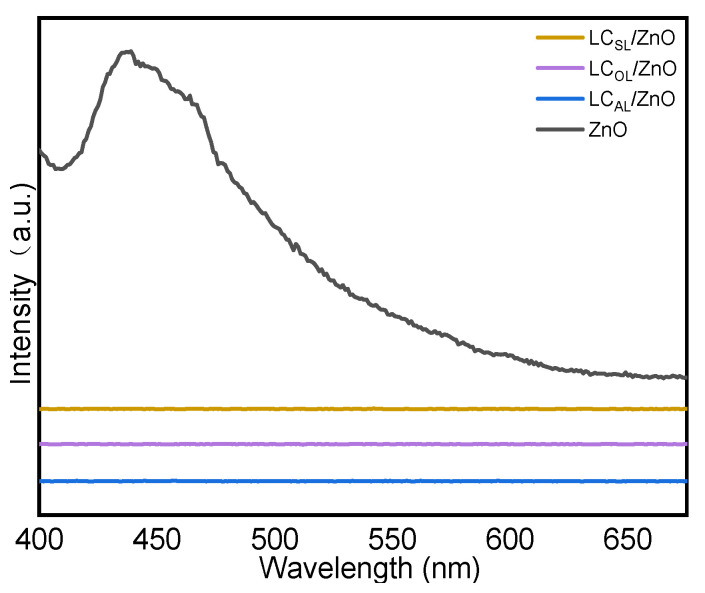
PL spectra of ZnO and LC_AL_/ZnO, LC_OL_/ZnO, and LC_SL_/ZnO.

The photoluminescence (PL) spectra of the ZnO and various ZnO-doped lignin-based carbons supports are shown in Figure 6. The ZnO showed a relatively high and broad PL peak around 450 nm, which indicated that the ZnO contain photogenerated electrons (e^−^) and holes (h^+^). Nevertheless, photogenerated electrons and holes sutures were highly likely to recombination. On the other hand, the various ZnO-doped lignin-based carbons were inferior to the PL strength of ZnO. On the contrary, the PL peak of various ZnO-doped lignin-based carbons at the same wavelengths decreases dramatically and even disappears, which was explained by a sharp decrease in the probability of recombination of photogenerated e^−^ and holes, and this result may be attributed to the significant improvement in photogenerated e^−^ separation and migration efficiency [43]. It also contributes to the photocatalytic reaction activity.

**Figure 7:**
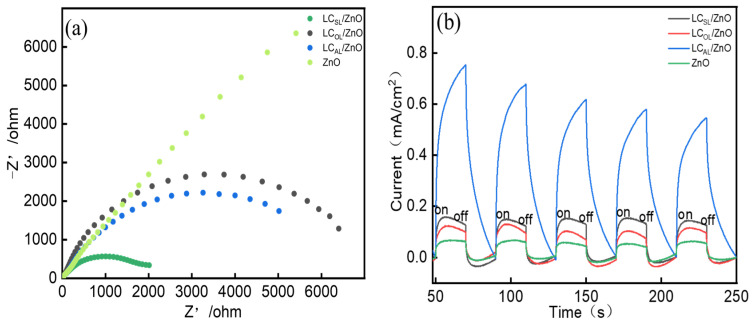
EIS (**a**) and I−T (**b**) curves of ZnO, LC_AL_/ZnO, LC_OL_/ZnO, and LC_SL_/ZnO with a bias voltage at 0.5 V.

The process of charge migration and separation of photogenerated e^−^ and holes deserves to be further investigated. Figure 7a shows that the trajectory radius of ZnO on the Nyquist curve was more diminutive than LC_AL_/ZnO, LC_OL_/ZnO, and LC_SL_/ZnO, which indicates that the interfacial charge transfer efficiency of ZnO was worse than that of the various ZnO-doped lignin-based carbons; the various ZnO-doped lignin-based carbons had a lower resistance, which was more favorable for the separation of photogenerated e^−^ and holes. It was consistent with the aforementioned photoluminescence spectra. Of these, LC_SL_/ZnO was the most prominent. The photoresponse cycle IT curves of the catalyst samples under five intermittent light irradiations are shown in Figure 7b. Photocurrent intensity reflects the efficiency of photogenerated charge separation and migration. The LC_AL_/ZnO, LC_OL_/ZnO, and LC_SL_/ZnO had significantly stronger photocurrents than the ZnO when the light was turned on, which indicated again that the efficiency of forming and migration of photo-induced charge carrier in the various ZnO-doped lignin-based carbons was much higher than that in the ZnO. The LC_SL_/ZnO had the strongest photocurrent, which meant that it had the highest photogenerated charge carrier efficiency. That might be because the different lignin carriers were tightly bound to the ZnO, which contributed to the improved photocatalytic activity compared to the ZnO.

### 2.3. Photocatalytic Activity of Various Prepared Catalysts

**Figure 8:**
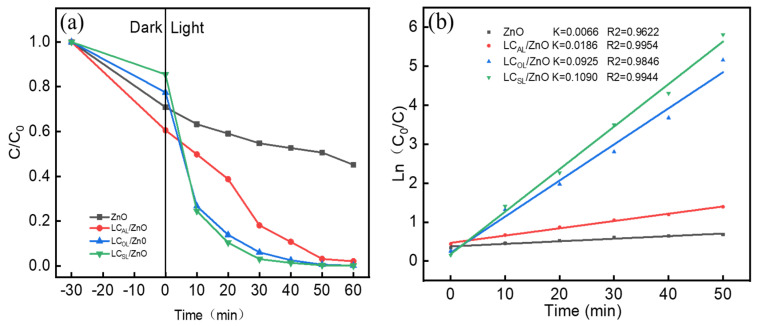
(**a**) MO degradation curves over ZnO and LC_AL_/ZnO, LC_OL_/ZnO, and LC_SL_/ZnO and (**b**) reaction kinetic curve.

The degradation of organic dyes was tested under simulated sunlight irradiation to further understand the photocatalytic performance of the prepared various ZnO-doped lignin-based carbons. During the experiment, the adsorption–desorption equilibrium curve of the shade was calculated preferentially. And as shown in Figure 8a, the curve data indicated that dye adsorption reached saturation within 30 min. The degradation rate of MO over the various ZnO-doped lignin-based carbons were significantly higher than that of the ZnO. After 30 min of light exposure, LC_OL_/ZnO and LC_SL_/ZnO almost completely degraded the MO, and the degradation rate of MO over LC_AL_/ZnO also reached 90% within 60 min. But for pure ZnO, the degradation rate of MO over ZnO also reached only 64.9% within 60 min. Figure 8b shows the pseudo-primary kinetics of MO photodegradation by the ZnO and various ZnO-doped lignin-based carbons. The reaction rates of MO photodegradation catalyzed by LC_AL_/ZnO, LC_OL_/ZnO, and LC_SL_/ZnO were 0.0187, 0.0925, and 0.1090, respectively; they were 2.8 times, 14.0 times, and 16.5 times stronger than that catalyzed by pure ZnO, which indicated again that the combination of ZnO and various lignin-based carbon was beneficial for MO photocatalytic degradation.

To fully understand the industrial value of ZnO and ZnO-doped lignin-based carbon, we also further studied the effects of solution pH and catalyst dosage on MO photocatalytic degradation. As shown in Figure S1a–c, the various ZnO-doped lignin-based carbons displayed different sensitivities to solution pH. For example, compared to LC_AL_/ZnO, pH has less effect on the catalytic efficiency of MO by LC_OL_/ZnO and LC_SL_/ZnO. The best degradation effect of LC_OL_/ZnO and LC_SL_/ZnO was around pH = 12.0. Figure S1d–f displays the effect of catalyst dosage on MO degradation, and it was clear that the influence of catalyst dosage on MO degradation was more obvious than that of solution pH. For example, with the increase in the loading of catalyst, the degradation rate was faster and the time required to reach degradation equilibrium became shorter.

**Figure 9:**
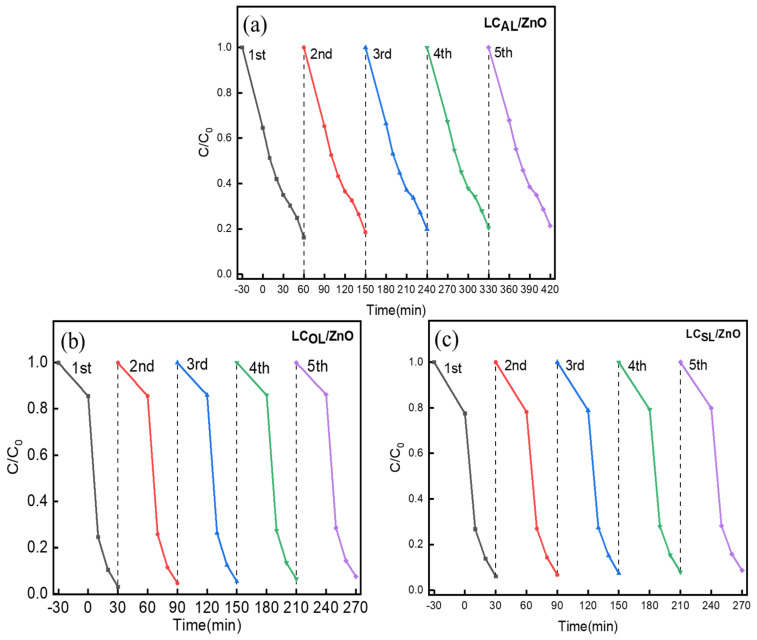
Multiple cycle experiments of LC_AL_/ZnO (**a**), LC_OL_/ZnO (**b**), and LC_SL_/ZnO (**c**) for MO photodegradation.

After understanding the photodegradation activity of various ZnO-doped lignin-based carbons, the exploration of their stability was the next goal, and five consecutive cycles of photodegradation of MO were carried out by the various ZnO-doped lignin-based carbons. The results of the experiment showed that the catalytic efficiency of various ZnO-doped lignin-based carbons only varied little and displayed good stability. The degradation efficiency of various ZnO-doped lignin-based carbons were reduced by about 5.0% in Figure 9a–c. They were indicating that the incorporation of different lignin-based carbon could slow down the photochemical corrosion process of ZnO. Therefore, the addition of lignin carbon base as a carrier not only improved the degradation efficiency, but also made it more stable. The above results showed that the ZnO-doped lignin-based carbon had excellent development prospects in the degradation of dye wastewater due to its exceptional stability.

**Figure 10:**
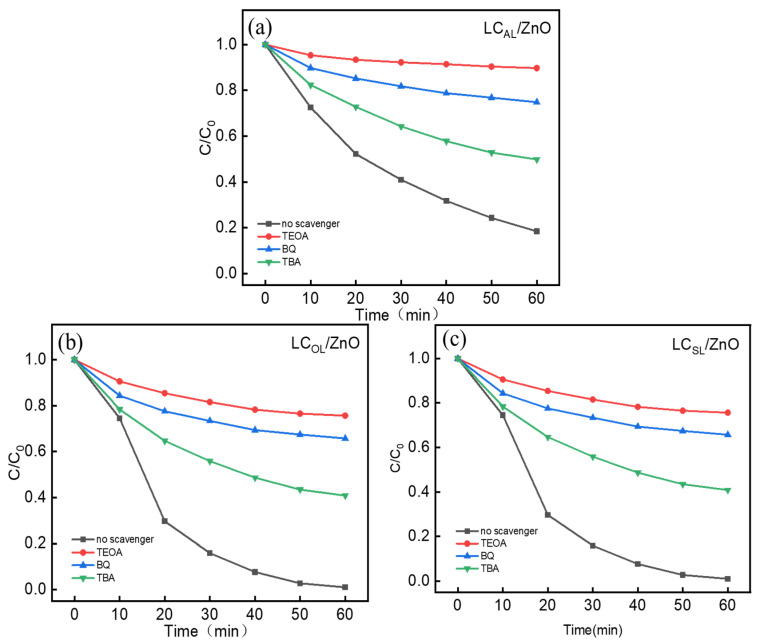
Effect of different trapping agents on the degradation of MO of LC_AL_/ZnO (**a**), LC_OL_/ZnO (**b**), and LC_SL_/ZnO (**c**).

To elucidate the contribution of various reactive radicals in the catalytic degradation of MO by the various ZnO-doped lignin-based carbons, scavenging experiments were conducted by adding various free radical trapping agents into the reaction system [44]. The trapping agents TEOA, BQ, and TBA were added to restrain photogenerated h^+^, hydroxyl radicals (·OH), and superoxide radicals (·O_2_^−^). As shown in Figure 10, the photodegradation efficiency of which were added TEOA, BQ, and TBA decreased exponentially compared to the solution without the added capture agent, indicating that the photogenerated h^+^, hydroxyl radicals (·OH), and superoxide radicals (·O_2_^−^) were photocatalytic active groups that played a key role in photodegradation of MO. Among them, the most obvious effect was TEOA; MO could hardly be photodegraded after adding TEOA in Figure 10, which indicates that h^+^ was the most vital active group in MO photocatalytic degradation.

In view of above active groups scavenging experiments, in this part, the degradation mechanism of MO by LC_SL_/ZnO were further analyzed and verified.

LC_SL_/ZnO + h*ν* → ZnO (h^+^) + LC (e^−^), (2)

O_2_+ e^−^ → ·O_2_^−^, (3)

H_2_O/OH^−^ + h^+^ → ·OH (4)

MO+ ·O_2_^−^ or h^+^ or ·OH → H_2_O and CO_2_ principally (5)

**Figure 11:**
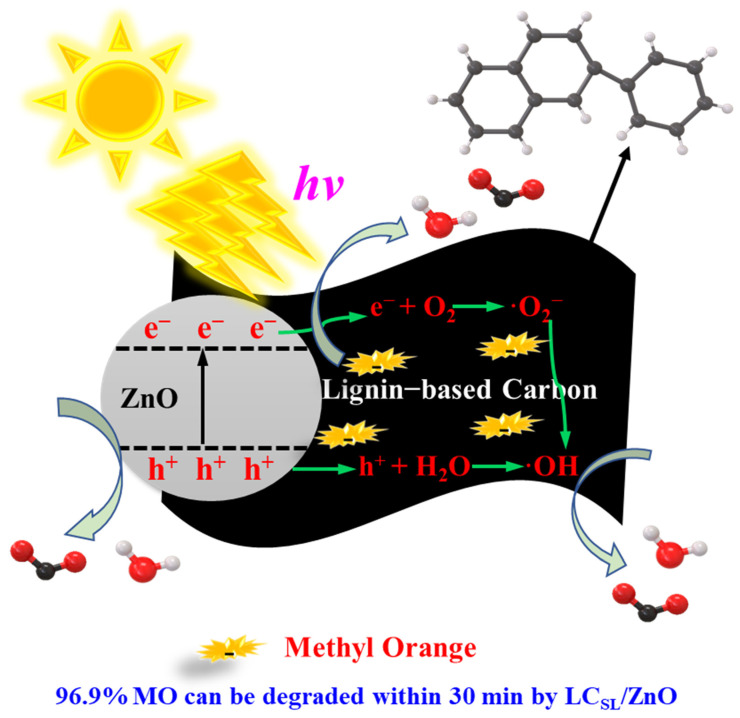
Catalytic degradation mechanism of MO by lignin-based carbon/ZnO composites.

As shown in Figure 11, due to the irradiation of simulated sunlight, electrons (e^−^) attached to the ZnO would migrate from the original valence band (VB) to the conduction band (CB). Photogenerated electrons and holes would be created from this process. They generated on ZnO surface and could be tightly bound by contact with each other and then transferred to lignin-based carbon nano-sheets to effectively separate them [45,46]. In addition, molecular oxygen is adsorbed on lignin-based carbon react with photogenerated electrons to produce superoxide ions (radical ·O_2_^−^). Both accessible radical ·OH and free radical ·O_2_^−^ could degrade MO due to their excellent oxidizing properties. On the other hand, holes also could directly degrade MO that was absorbed on the surface of ZnO.

## 3. Experimental Section

### 3.1. Chemicals and Reagents

Alkali lignin (AL) was purchased from the website of the energy and chemical industry (Guangzhou, China), organosolv lignin (OL) was prepared by acid precipitation, sulfonated lignin (SL) was purchased from China Shanghai Aladdin Biochemical Technology Co., Ltd. Sodium oxalate (Na_2_C_2_O_4_, >99.9%) was purchased from Macklin. Zinc nitrate hexahydrate (Zn(NO_3_)_2_·6H_2_O) was purchased from China Nanjing Chemical Reagent Co., Ltd. Methyl orange (MO), tert-butanol (TBA, (CH_3_)_3_COH), triethanolamine (TEOA, C_6_H_15_NO_3_) and benzoquinone (BQ, C_6_H_4_O_2_) were all from China Shanghai Maclean Biochemical Co., Ltd.

### 3.2. Preparation of Various ZnO-Doped Lignin-Based Carbons

**Figure 12:**
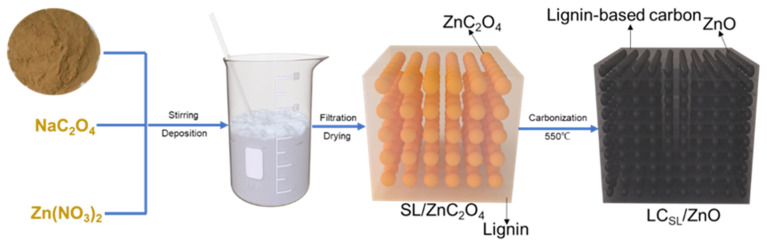
Schematic diagram of LC_SL_/ZnO composite preparation.

Firstly, Na_2_C_2_O_4_ (6.7 g) solution was neutralized with 250 mL of deionized water and then Zn(NO_3_)_2_·6H_2_O (14.87 g) was dissolved in the same volume of deionized water. The rotor was added to the solution and placed on a liquid stirrer, and then the Zn(NO_3_)_2_ solution was dropped into it, and the dispersed ZnC_2_O_4_ mixture was obtained by continuous stirring. Subsequently, 0.5 g of AL OL and SL power were added to the said compound and stirred for half an hour. Then, the mixture was filtered, and the ZnC_2_O_4_/AL ZnC_2_O_4_/OL and ZnC_2_O_4_/SL precursor were heated at 60 °C for 6 h, and the solid obtained above was ground into powder and allowed to be calcined at a high temperature of 550 °C for 120 min in a high temperature carbonization furnace (OTF-1200X) filled with nitrogen to obtain a carbonized solid specimen. Various ZnO-doped lignin-based carbons (ZnO-Doped Lignin-Based Carbons) were prepared. The ZnO composites of the above three different lignin carriers were only different in the type of added lignin, and the other processes were basically the same, so the flow chart of LC_SL_/ZnO is highlighted in Figure 12.

### 3.3. Characterization Methods

The micromorphology and microstructure of the prepared specimens were examined by Scanning Electron Microscope (SEM, Regulus 8100, Hitachi, Tokyo, Japan) and Transmission Electron Microscope (TEM, JEM-1400F, JEOL, Tokyo, Japan). The ZnO, LC_AL_/ZnO, LC_OL_/ZnO, and LC_SL_/ZnO were placed in anhydrous ethanol and allowed to disperse homogeneously by using an ultrasonic device, dispersed samples prepared dripped onto a silicon chip and was allowed to dry in a ventilated area. Finally, the prepared specimens were attached to the specimen stage with conductive adhesive for the testing and analysis of test results. When TEM specimens were measured, the silicon chip was changed into copper mesh, and the remaining operations were basically the same. The composition and crystallinity of pure ZnO and various ZnO-doped lignin-based carbons were collected by multifunctional horizontal X-ray diffractometer (XRD, Rigaku, XRD Ultima IV, Tokyo, Japan), in which the wavelength of Cu Kα was 0.1541 nm and the power of X-ray generator was 3 KW. Catalyst composition, structure, and relative content were recorded on the composition, structure and relative content of the catalysts were further characterized by laser Raman spectroscopy at a wavelength of 633 nm.(DXR532, Themor, MA, USA). X-ray photoelectron spectroscopy (XPS, Axis Ultra, Kratos Analytical Ltd., Stretford, UK) was employed to study elemental composition of the catalyst surface. Nitrogen adsorption–desorption was employed to study the specific surface area and pore size of the specimens. The optical properties of ZnO and various ZnO-doped lignin-based carbons were studied by UV-visible diffuse reflectance spectroscopy (UV-1780, Shimadzu, Kyoto, Japan) and photoluminescence (PL) spectroscopy of transient steady-state fluorescence spectrometer. The photocurrent time (I-T) current was recorded at 0.5 mV bias potential during 5-cycle light on and off. The devices’ electrochemical impedance spectroscopy (EIS) curves at the frequency range between 105 and 10−1 Hz were recorded under dark conditions, under a bias voltage of 5 mV. All of the photochemical and electrochemical measurements were performed in Na_2_SO_4_ (0.1 M) solution as electrolyte at ambient temperature. FTO glass (10 × 10 × 2.2) was sonicated and rinsed with deionized water [47]. The specimens were then uniformly distributed on clean FTO glass using a low rotation process, and then the testing began.

### 3.4. Photocatalytic Experiment

The photodegradation of MO was employed to evaluate the photocatalytic activity of ZnO and various ZnO-doped lignin-based carbons. MO solution (15 mg·L^−1^, 100 mL) and the prepared specimens (50 mg) were irradiated with simulated sunlight (300 W xenon lamp) in a photochemical reaction instrument (CME-Xe300F, Beijing, China) under stirring. The mixture was first left in dark environment for 30 min to maximize their adsorption equilibrium. During the reaction, 2 mL mixture was taken out every 10 min, and the catalyst in the mixture was removed by an experimental filter membrane. The concentration of MO solution with photocatalyst released was measured by UV-vis spectrophotometry through absorbance at 464 nm (it was best to ensure that the initial absorbance of MO was about 0.5 by proper dilution in the experiment). The degradation rate of MO is expressed as C/C_o_. Among them, C is the MO concentration calculated in real-time from absorbance, and C_o_ is the initial concentration of the MO solution.

In addition, dark reaction process without the participation of light was carried out, which helped to exclude the effect of adsorption on the catalytic effect. The active groups were researched by dissolving various trapping agents. Triethanolamine (TEOA), tert-butyl alcohol (TEA), and benzoquinone (BQ) were employed to capture photogenerated holes (h^+^), hydroxyl radicals, and superoxide radicals, respectively. Divided into three sets of experiment, 500 μL TEA, 5 mg BQ, or 5 mg TEOA were added to the MO solution (15 mg·L^−1^, 100 mL) containing catalyst (50 mg) in order to identify the reactive groups that have the greatest influence on the catalytic degradation of MO.

## 4. Conclusions

The ZnO nanoparticles and lignin-based carbon were tightly combined during the carbonization process, and finally led to the formation of uniform LC/ZnO composites. All the various ZnO-doped lignin-based carbons prepared in this work had strong efficiency for photogenerated electrons and holes separation and migration, which was mainly due to the outstanding surface contact between the ZnO and carbon support. The photocatalytic activity of ZnO with various lignin-based carbons support increased exponentially compared with that of the pure ZnO, especially with the carbon support prepared from sulfonated lignin. The MO (15 mg/L) could be almost completely degraded by LC_SL_/ZnO composite within 30 min in a large pH range from 2 to 12. Scavenging experiments confirmed that h^+^, ·O_2_^−^, and ·OH were the main active species in the photocatalytic degradation of MO, among which h^+^ plays the most important role. In addition, the cycling experiments indicated that all prepared LC/ZnO composites had the good stability. Especially for LC_SL_/ZnO, the degradation rate of MO was reduced by only five percent after five reproducible experiments.

## Data Availability

Data are contained within the article and Supplementary Materials.

## Supplementary Materials

The following supporting information can be downloaded at: https://www.mdpi.com/article/10.3390/molecules29081817/s1.
