# Supplementary material for: Efficient Catalytic Degradation of Methyl Orange by Various ZnO-Doped Lignin-Based Carbons
Source: Molecules. 2024 Apr 17;29(8):1817. doi: 10.3390/molecules29081817 (PMC11052459; doi:10.3390/molecules29081817)
Supplement: Supplementary file 1 [file molecules-29-01817-s001.zip › molecules-2947400-supplementary.pdf]

Efficient catalytic degradation of methyl orange by Various ZnO-doped lignin-based carbons

Zhihao Tang [1], Yonggang Yang [2] and Weiqi Wei [1, 2, *]

[1] Jiangsu Co-Innovation Center of Efficient Processing and Utilization of Forest Resources,

Nanjing Forestry University, Nanjing 210037, China ; tangzhihao@njfu.edu.cn

[2] School of Environmental & Resource Science, Shanxi University, Taiyuan 030006,

China; m18352353192@163.com

* Correspondence: wqwei@njfu.edu.cn (Weiqi Wei)

Table S1. The specific surface area, total pore volume and average pore diameter of ZnO and LC_{AL}/ZnO, LC_{OL}/ZnO and LC_{SL}/ZnO.

**Sample Name**	**BET surface area (m^2/g)**	**Total pore volume (cm^3/g)**	**Average pore diameter (nm)**
ZnO	24.8436	10.061295	28.2653
LC_{AL}/ZnO	52.5159	10.114030	16.3155
LC_{OL}/ZnO	64.4977	20.134970	17.4985
LC_{SL}/ZnO	79.6220	30.042036	6.8782

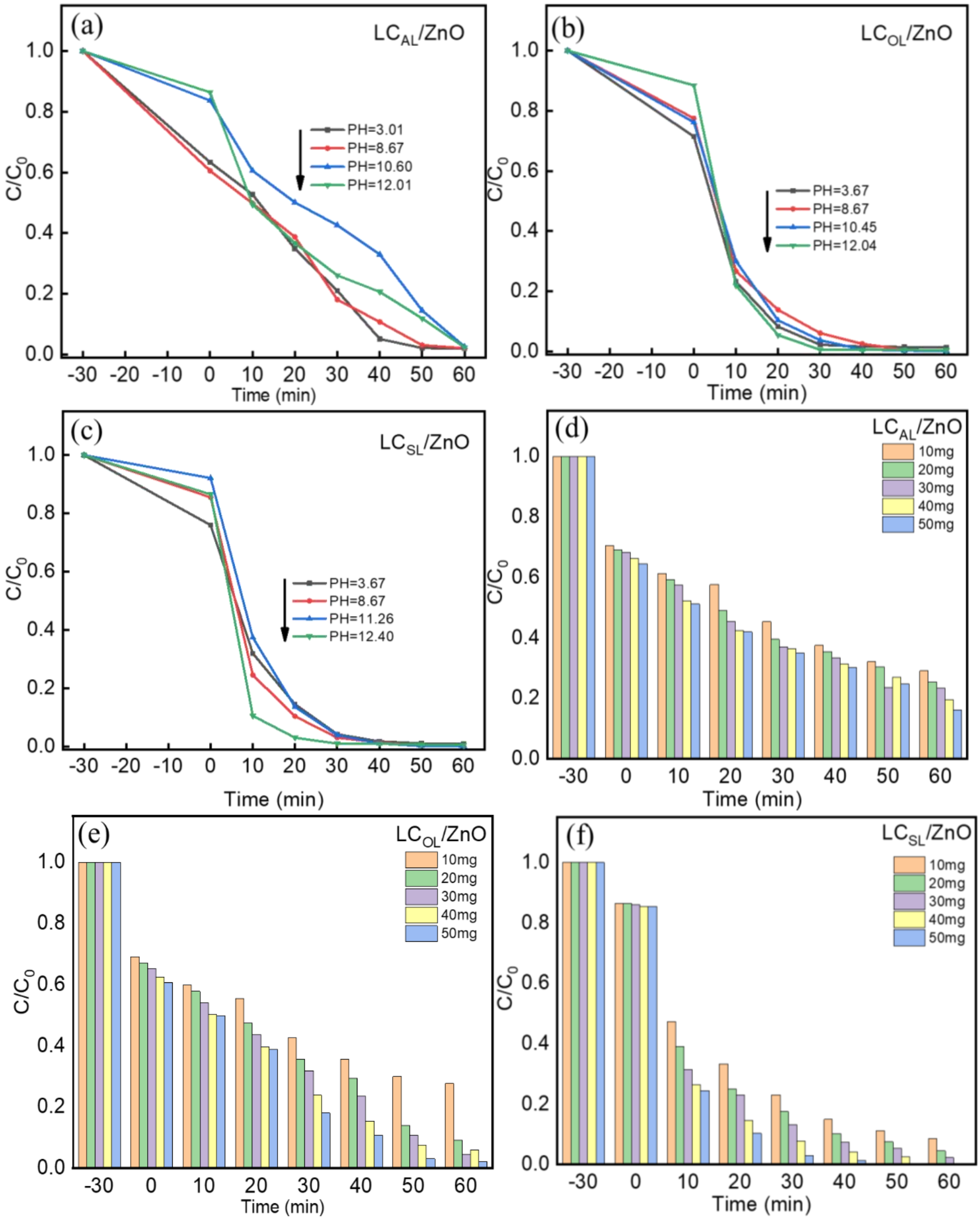

Figure S1. The effect of pH (a b & c) and catalyst dosage (d e & f) on MO photodegradation.
